# *Big Pharma* (The Play)

**DOI:** 10.1371/journal.pbio.0050104

**Published:** 2007-04-17

**Authors:** Lisa Bero

Anyone who has experienced the assault of the pharmaceutical industry's marketing campaigns would appreciate Jennifer Berry's one-person play *Big Pharma: The Rise of the Anti-Depressant Drug Industry and the Loss of a Generation*. Since the mid-1990s, spending on drug promotion has grown steadily, reaching $21 billion in 2002. Berry explores the fallout of this expanded marketing blitz through the eyes of its masterminds, unwitting (and complicit) abettors, and victims through her portrayal of an advertising executive, a physician, and women and children who are prescribed heavily marketed antidepressants.

A primary target of the pharmaceutical industry, physicians receive not just advertising materials but office visits from drug representatives. Berry's physician, depicted as a pawn of the pharmaceutical industry, gratefully accepts the free drug samples, the free lunches, and the pharmaceutical industry–sponsored trips to tropical islands. In fact, the pharmaceutical industry woos physicians with educational dinners, honoraria for participating in conference calls, consulting fees for participating in speakers' bureaus, research funding, and payments to write scientific publications. And physicians act as agents of the pharmaceutical industry in many ways, such as giving talks that favor a company's product, participating in clinical trials that increase physicians' exposure to a new drugs or new indications for old drugs, and publishing research articles that are financed and, in fact, written by pharmaceutical company employees [1].[Fig pbio-0050104-g001]

**Figure pbio-0050104-g001:**
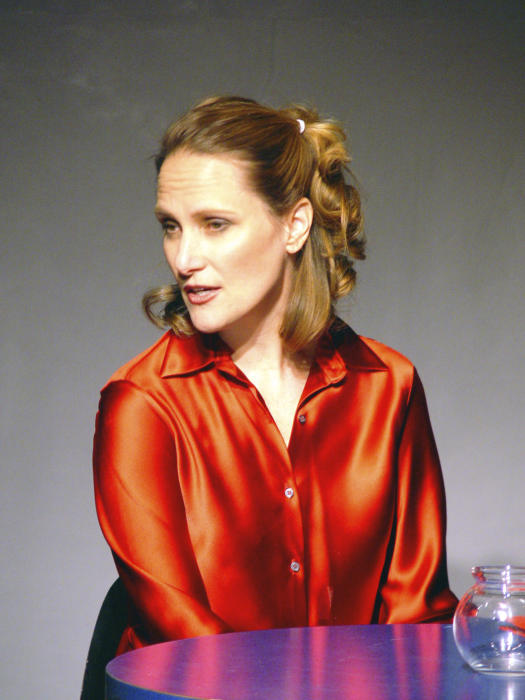


In addition to reaching physicians directly, the industry promotes consumer familiarity with particular drugs. In 2002, almost $3 billion of the industry's drug promotion budget was spent on direct-to-consumer advertising [2], with advertising venues spanning from print media to radio, television, and the Internet [3]. In her play, Berry notes that direct-to-consumer drug advertising skyrocketed in the United States after a 1997 change in the Food and Drug Administration guidelines for advertising. She also gives a nod to the nearly incomprehensible patient information that must be provided with each ad: “We tell them to buy it, then tell them why not to buy it.” And, the “why not” is presented in an illegible font in print ads, or high-speed chatter for television and radio.

Less obvious marketing strategies include using opinion leaders, and sponsoring education programs, scientific research, publications, and professional meetings. The pharmaceutical industry has also extended its influence to clinical trial administration, research design, regulatory lobbying, physician and patient education, drug pricing, pharmacy distribution, and drug compliance [4].

There is a wide range of evidence suggesting that all of these marketing strategies influence physician prescribing and affect patient attitudes and behavior. Interactions with the pharmaceutical industry increase the likelihood of physicians prescribing inappropriately or making formulary requests for the company's product [5–9]. Yet health professionals at all levels of training tend to believe that they are not influenced by the drug industry [10–13]. For instance, research suggests that most health-care professionals think information from drug companies is biased, but many think it is useful nonetheless. With regard to direct-to-consmer advertising, most doctors are opposed to it, primarily because it negatively impacts the doctor–patient relationship and pressures doctors to prescribe drugs they might not otherwise use [2,14].

Empirical evidence regarding direct-to-consumer advertising shows that it acts as promotion rather than education, often includes inaccurate, misleading, or unbalanced information, frequently includes emotional appeals to anxiety about illness, underplays risks, omits mention of costs, and tends to promote the “medicalization” of normal health and minor ailments [15–17]. And consumers tend to have positive views of drug advertisements, show awareness of advertisements, and exhibit a willingness to discuss or request from their doctor a drug they have seen advertised [15,18,19]. Pointedly, Berry's physician fears his patients will sue if he doesn't prescribe the drugs they ask for.

The notion that the pharmaceutical industry creates diseases or “disorders” when none exist surfaces as a major theme of the play. Berry's advertising executive proudly displays direct-to-consumer advertisements for antidepressants. The executive explains that she “can't just say ‘buy drugs,’” so the advertisers develop a campaign that preys on women with societal problems, such as stress or homelessness. Clearly, pharmaceutical marketing practices impact definitions of social concern, health, and illness. For example, Moynihan and Cassels [16] and Moynihan and Henry [17] discuss how the expansion of pharmaceutical marketing practices leads to different forms of disease mongering: aspects of ordinary life, such as menopause, are treated as medical conditions; mild problems are portrayed as serious illnesses, as occurred with the drug company–sponsored promotion of irritable bowel syndrome; and risk factors, such as high cholesterol and osteoporosis, are framed as diseases. One example touched on in Berry's play is the condition of “social anxiety disorder,” which is diagnosed in a poor, unemployed woman who is having trouble with job interviews [16]. Berry dramatizes the solution—prescribe an antidepressant rather than find a job—by solving every character's problem with a drug prescription.

And it is Berry's patient characters that have the most impact. The patients are portrayed as victims not only of a profit-seeking pharmaceutical industry, but of society in general. Berry describes the pharmaceutical industry's target market as women who are subjected to sexism, racism, and social inequalities. She describes how the pharmaceutical industry has transformed these societal problems into medical “disorders” that can be treated with a pill—in this case an antidepressant. Children who dare to be different or energetic are also labeled with a disorder. Berry's soliloquy poignantly describes how antidepressants transform vibrant individuals into quiet, passive people with a lack of affect. Her description of the side effects of antidepressants is completely accurate. Unfortunately, the play does give the impression that most women are not strong enough to help themselves. But, of course, this is what the pharmaceutical industry is counting on.

My favorite part of the play is when Berry acts one half of a conversation with an elderly woman who lived through the Great Depression. She describes how everyone was sad, but, together, people survived. Berry contrasts this to our current era of the “anti-Depression,” when unhappiness is considered a disease that must be eradicated.

I have to admit that Berry's performance left me a bit depressed. I was depressed by the fact that the pharmaceutical industry manages to take advantage of societal problems to market their products and increase their profits. At the same time, the industry's tactics were exposed and the audience had a chance to think about how to channel unhappiness to confront our real problems and not just treat the symptom. After the play, I did have an urge to go home and be sad for a while...but not to pop any pills.
